# *In silico* evaluation of limited sampling strategies for individualized dosing of extended half-life factor IX concentrates in hemophilia B patients

**DOI:** 10.1007/s00228-021-03173-2

**Published:** 2021-10-15

**Authors:** T. Preijers, M. W. F. van Spengler, K. Meijer, K. Fijnvandraat, K. Fischer, F. W. G. Leebeek, M. H. Cnossen, R. A. A. Mathôt

**Affiliations:** 1 Hospital Pharmacy-Clinical Pharmacology, Amsterdam University Medical Center, Amsterdam, The Netherlands; 2 Department of Pediatric Hematology, Academic Medical Center Amsterdam, Amsterdam, The Netherlands; 3 Department of Hematology, University of Groningen, University Medical Center Groningen, Groningen, The Netherlands; 4 Van Creveldkliniek University Medical Center Utrecht, Utrecht, The Netherlands; 5 Department of Hematology, Erasmus University Medical Center, Rotterdam, The Netherlands; 6 Department of Pediatric Hematology, Erasmus University Medical Center, Sophia Children’s Hospital, Rotterdam, The Netherlands

**Keywords:** Hemophilia B, Coagulation factor IX, Computer simulation, Pharmacokinetics, Coagulation factor concentrates

## Abstract

**Purpose:**

Hemophilia B is a bleeding disorder, caused by a factor IX (FIX) deficiency. Recently, FIX concentrates with extended half-life (EHL) have become available. Prophylactic dosing of EHL-FIX concentrates can be optimized by assessment of individual pharmacokinetic (PK) parameters. To determine these parameters, limited sampling strategies (LSSs) may be applied. The study aims to establish adequate LSSs for estimating individual PK parameters of EHL-FIX concentrates using *in silico* evaluation.

**Methods:**

Monte Carlo simulations were performed to obtain FIX activity versus time profiles using published population PK models for N9-GP (Refixia), rFIXFc (Alprolix), and rIX-FP (Idelvion). Fourteen LSSs, containing three or four samples taken within 8 days after administration, were formulated. Bayesian analysis was applied to obtain estimates for clearance (CL), half-life (*t*_1/2_), time to 1% (Time_1%_), and calculated weekly dose (Dose_1%_). Bias and precision of these estimates were assessed to determine which LSS was adequate.

**Results:**

For all PK parameters of N9-GP, rFIXFc and rIX-FP bias was generally acceptable (range: −5% to 5%). For N9-GP, precision of all parameters for all LSSs was acceptable (< 25%). For rFIXFc, precision was acceptable for CL and Time_1%_, except for *t*_1/2_ (range: 27.1% to 44.7%) and Dose_1%_ (range: 12% to 29.4%). For rIX-FP, all LSSs showed acceptable bias and precision, except for Dose_1%_ using LSS with the last sample taken on day 3 (LSS 6 and 10).

**Conclusion:**

Best performing LSSs were LSS with samples taken at days 1, 5, 7, and 8 (N9-GP and rFIXFc) and at days 1, 4, 6, and 8 (rIX-FP), respectively.

## Introduction

Hemophilia B is an X-linked congenital bleeding disorder, caused by over 2100 different mutations in the factor IX (FIX) gene resulting in a factor IX deficiency. Patients with endogenous baseline FIX activity of 5 to 40%, 1 to 5%, and less than 1% of normal are classified as mild, moderate, and severe hemophilia B, respectively [1]. Severe and some moderate hemophilia B patients experience spontaneous bleedings in their joints and muscles [2]. Without adequate prophylactic or on-demand treatment, these bleedings lead to damage of joints and muscles, resulting in pain, immobilization, and potential long-term invalidity [3].

Currently, the standard of treatment aims to prevent and treat spontaneous and trauma-related bleedings by prophylactic replacement therapy with FIX concentrates [4]. In prophylaxis, FIX concentrates are dosed to obtain trough activity levels depending on the bleeding phenotype, but generally above 1%. The average terminal elimination half-life of the “standard” half-life (SHL) FIX concentrates lies between 18 and 24 h [5]. To achieve adequate prophylactic treatment, SHL-FIX concentrates are administered at least twice weekly [4]. Since 2016, three FIX concentrates with an extended half-life (EHL) have been approved by the European Medicines Agency: PEGylated FIX (N9-GP, Refixia®), FIX fused to the neonatal Fc receptor (rFIXFc, Alprolix®), and FIX fused with human albumin (rFIX-FP, Idelvion®) [6–8]. These EHL-FIX concentrates are produced using recombinant-DNA techniques and have been modified to reduce the rate of elimination and, hence, extend half-life up to fourfold [9]. It has been reported that prophylactic administration of EHL-FIX concentrates may decrease frequency of intravenous dosing [10, 11]. Moreover, due to the extended half-life, FIX activity levels are above the target value of 1% for longer periods using similar doses compared with the SHL-FIX concentrates [12]. Inversely, frequency of FIX peak levels will decrease, with a potential increase of bleeding [13].

Significant variability in pharmacokinetics (PK) between patients has been observed for EHL-FIX concentrates. Therefore, determination of an individual PK profile may be beneficial [11, 14–16]. The PK profile of a patient is determined by the individual’s PK parameters, e.g., clearance (CL), volume of distribution (V), and terminal half-life (*t*_1/2_). The values of these parameters can be used to individualize EHL-FIX concentrate dosing [17]. To accurately determine the individual PK parameters, the number and the timing of blood sampling must be well-determined [18].

Currently, it is not clear when blood sampling after EHL-FIX administration, e.g., frequency and timing, should be performed [19]. Although limited sampling strategies (LSSs) have been determined for SHL-FIX products [20], these LSSs may not be applicable to the EHL-FIX concentrates due to the differences in PK. Therefore, this study aims to establish limited sampling strategies (LSSs) to estimate individual PK parameter values for EHL-FIX concentrates by using in silico evaluation.

## Methods

In this study, concentration–time data was simulated *in silico* for three EHL-FIX products: N9-GP (Refixia, Novo Nordisk A/S, Denmark), rFIXFc (Alprolix, Swedish Orphan Biovitrum AB, Sweden), and rIX-FP (Idelvion, CSL Behring GmbH, Germany) [14–16]. Monte Carlo simulations were performed using population PK models, as constructed for the three compounds (see below). Subsequently, the predictive performance of 14 LSSs was evaluated for these models. Individual PK parameter estimates were obtained for every LSS using Bayesian analysis. From this analysis, individual PK parameter estimates were obtained and were compared with simulated individual PK parameter values to determine the predictive performance of the LSSs.

### Population simulation

R (R Core Team [21], version 3.4.1) [21] was used to simulate a dataset of 10,000 virtual patients with varying weight, all of whom received a single dose of 50 IU kg^−1^ rounded to the nearest multiple of 250 IU corresponding to the minimum vial content. Infusion duration ranged randomly between 2 and 5 min. The body weights of the patient populations used to construct the published population PK models for N9-GP, rIX-FP, and rFIXFc ranged from 56 to 90 kg, 11 to 132 kg, and 45 to 187 kg, respectively [14–16]. Therefore, body weights were simulated for all virtual patients, ranging from 11 to 187 kg, representing the combined studied body weight ranges from the populations in the literature. Subsequently, a selection from these 10,000 virtual patients was taken for each EHL-FIX product, based on the three investigated body weight ranges, as reported in the respective publications.  

### Pharmacokinetic simulation

To simulate concentration–time curves for the patients from the simulated datasets, Monte Carlo simulations were performed. In a PK Monte Carlo simulation, individual PK parameters are generated for each patient using the values from the population PK parameters and their corresponding inter-patient variability (IIV). Using the individual PK parameters, concentrations can be calculated for each desired time point. In Monte Carlo simulation, the residual variability is also taken into account, from which random errors are generated. These errors allow mimicking intra-patient variability, time entry discrepancies of dosing or blood sampling, and errors in the assay used to measure the FIX activity. Ultimately, the simulated residual variability is added to the simulated concentration to yield a simulated observation (i.e., FIX level measurement with assay error and intra-patient variability).

In this study, concentration–time curves, or individual PK profiles, were obtained using NONMEM v7.4.1 (ICON Development Solutions, Ellicott City, Maryland, USA) software [22]. The models published in the literature were used for these simulations (Table 1) [14–16]. The data used to develop these population PK models were collected from severe and moderate hemophilia B patients. Population PK parameters were described in terms of CL, inter-compartmental clearances (Q, Q2, Q3), and the volumes of distribution from the different compartments (V1, V2, V3). In the N9-GP model, the population PK parameters were normalized by the body weight of the patients and are specified in units per kg body weight. In the population PK model for rFIXFc, population PK parameters CL and V1 were allometrically scaled using separate exponents and a median body weight of 73 kg. In the population PK model for rIX-FP, the population PK parameters CL, V1, and V2 were allometrically scaled to a reference body weight of 70 kg and separate exponents were applied. Furthermore, the rIX-FP model used a weight-adjusted dose factor to scale V1 by the amount of the administered dose. For all population PK models, the population PK parameters CL and V1 contained inter-patient variability. Additionally, inter-occasion variability (IOV) was described for CL and V1 in the population PK model for rFIXFc, which was taken into account when simulations were performed. Moreover, complete washout was assumed and no endogenous baseline level was simulated.

**Table 1:** Population PK parameter estimates from published models

	N9-GP^a^		rFIXFc^b^		rIX-FP^c^	
	Estimate	RSE (%)	Estimate	RSE (%)	Estimate	RSE (%)
Structural model						
Clearance (CL; mLh^−1^)	0.684^*^	4.6	239	-	57	2.7
Volume of central compartment (V1; mL)	73.9^*^	4.8	7140	-	6480	3.2
Distribution CL to compartment 2 (Q(2); mLh^−1^)	0.614^*^	35.2	167	-	29	36.4
Volume of compartment 2 (V2; mL)	15.6^*^	11.8	8700	-	1580	12.1
Distribution CL to compartment 3 (Q3; mLh^−1^)	-	-	3930	-	-	-
Volume of compartment 3 (V3; mL)	-	-	3990	-	-	-
Baseline FIX level	-	-	-	-	0.0106	11.6
Body weight exponent on CL	-	-	0.436	-	0.53	9.3
Body weight exponent on V1	-	-	0.396	-	0.79	6.6
Body weight exponent on V2	-	-	-	-	0.79	6.6
Weight-adjusted dose exponent on V1	-	-	-	-	0.38	16.9
Inter-individual variability (%CV^d^)						
IIV on CL	16.8	-	17.8	-	21.1	22.0
IIV on V1	18.7	-	21.7	-	25.9	30.2
IIV on V2	-	-	46.1	-	-	-
IIV on V3	-	-	37.7	-	-	-
IIV on Q(2)	127.3	-	35.9	-	-	-
Correlation between CL and V1 (%)	16.1	17.5	75.6	-	-	-
IIV on baseline	-	-	-	-	39.5	41.5
Inter-occasion variability (%CV)						
IOV CL	-	-	15.2	-	-	-
IOV V1	-	-	17.4	-	-	-
Residual variability						
Additive residual variability (SD; IUmL^−1^)	0.000267	41.6	0.0024	-	0.0066	27.6
Proportional residual variability (%CV)	6.47	56.1	10.6	-	18	11.4
Population half-life						
*t*_1/2_ (h)	94.3	-	79	-	108.3	-

*RSE* relative standard error, *CV* coefficient of variation, *SD* standard deviation

^*^kg^−1^

^a^Diao et al. [15]

^b^Zhang et al. [16]

^c^Iorio et al. [17]

^d^Calculated as √(e^^Var^ − 1) × 100%

### Limited sampling strategies

Prior to the simulations, a total of 14 LSSs, with samples taken between 10 min and 8 days after administration, were formulated (Table 2). An optimal LSS leads to accurate estimations with minimal sampling. Based on the number of compartments in the applied population PK models, LSSs were evaluated with a minimum of three to four blood samples, taken on specific days. For each LSS, samples were taken on different days to determine the appropriate moments for sampling. A sample taken on the first day can be taken shortly after administration, making an additional hospital visit unnecessary. Therefore, a sample taken on the day of administration was included in every LSS. Moreover, sampling windows were chosen during working hours, to accommodate patient and treating physician. For each LSS, simulated concentrations were taken at random from the corresponding sampling days. Hereby, a dataset was generated for each LSS, containing only the FIX concentrations from the sampling days as specified for the LSS. For LSSs with two samples within the same sampling window, the time of sampling was at least 30 min apart.

**Table 2:** Limited sampling strategies evaluated using Bayesian analysis

LSS	Day 1	Day 2	Day 3	Day 4	Day 5	Day 6	Day 7	Day 8	Total no. of samples
	0.167–3 h	24–32 h	48–56 h	72–80 h	96–104 h	120–128 h	144–152 h	168–176 h	
1	x	x	x	x					4
2	x		x		x		x		4
3	x			x	x		x		4
4	x			x		x		x	4
5	x				x		x	x	4
6^*^	x	xx	x						4
7^*^	x		xx	x					4
8^*^	x			xx	x				4
9^*^	x				xx	x			4
10	x	x	x						3
11	x		x		x				3
12	x			x				x	3
13	x		x			x			3
14	x				x			x	3

^*^Limited sampling strategy (LSS) with two measurements on the same day, separated by a minimum of 30 min.

### Bayesian analysis

In Bayesian analysis, population PK parameters are taken as a priori information. This population information is used in combination with information concerning the individual patient (e.g., observations, dosing information, and body weight), to determine the individual PK parameters that most likely describe the concentration–time curve from that individual. Therefore, having more observations is similar to supplying more information and, provided that FIX level measurements were well-timed, improves the accuracy of these estimates. Furthermore, samples taken during specific sampling windows may be more important for predictive performance as compared with other sampling windows. For instance, if no sample is obtained at or near the peak FIX concentration, the observed FIX concentrations are likely to contain less information about the central volume of distribution (V1). Consequently, this may lead to a poor estimation of this individual PK parameter.

Bayesian analysis was performed with NONMEM software. This analysis yielded estimated values for the individual PK parameters, based on the simulated FIX concentrations from the LSS datasets. The patients having a FIX level below the lower limit of quantification (BLQ) were discarded from analysis, as a value BLQ does not allow precise estimation of the individual PK parameters in clinical practice.

### Assessment of predictive performance

To determine the performance of the LSSs, their ability to estimate clearance of the central compartment (CL), terminal elimination half-life (*t*_1/2_), time until 1% (Time_1%_), and the calculated weekly dose (Dose_1%_) was assessed. Time_1%_ and Dose_1%_ were calculated using the equations from Dubois et al. [23]. Dose_1%_ was defined as the dose required to yield a 1% FIX level 1 week after administration. The individual PK parameter values obtained from the Monte Carlo simulations were considered as the golden standard. The bias and precision of the estimation, concerning the true individual PK parameters being the golden standard, were imputed by the relative mean prediction error (rMPE) and the relative root-mean-square error (rRMSE), respectively, using the following equations:

$${rMPE}_{i}=\frac{1}{n}{\sum }_{j=1}^{n}\left(\frac{{\widehat{\theta }}_{ij}-{\theta }_{ij}}{{\theta }_{ij}}\right)\times 100\%,$$

$${rRMSE}_i=\sqrt{\frac1n{\textstyle\sum_{j=1}^n}\left(\frac{{\widehat\theta}_{ij}-\theta_{ij}}{\theta_{ij}}\right)^2\times100\%.}$$

Here, $i$ is the LSS number as shown in Table 2, $n$ is the number of patients, ${\widehat{\theta }}_{ij}$ is the individual PK parameter estimate for the $j$-th individual, and ${\theta }_{ij}$ is the true value of the individual parameter, taken from the Monte Carlo simulation. A negative or positive rMPE indicates a systematic underestimation or overestimation of the parameter, respectively. An rMPE between −5% and 5% was considered to be adequately accurate. Large values of the rRMSE in combination with an rMPE close to zero generally indicate large deviations from the true individual parameter values without a specific tendency for underestimation or overestimation. If an LSS had an rRMSE greater than 25%, it was considered inadequate for clinical practice.

Moreover, the difference between the golden standard and the estimates individual PK parameter value were described by the relative prediction error (rPE) and were calculated using the following equation:

$${rPE}_{ij}=\frac{{\widehat{\theta }}_{ij}-{\theta }_{ij}}{{\theta }_{ij}}.$$

Here ${\widehat{\theta }}_{ij}$ and ${\theta }_{ij}$ are the individual estimate and the true individual value of PK parameter $i$ of the $j$-th patient, respectively. The 95% ranges from the values of the relative prediction errors were visualized using boxplots. LSSs with a range between −30% and 30% for each parameter were deemed acceptable.

## Results

**Fig. 1:**
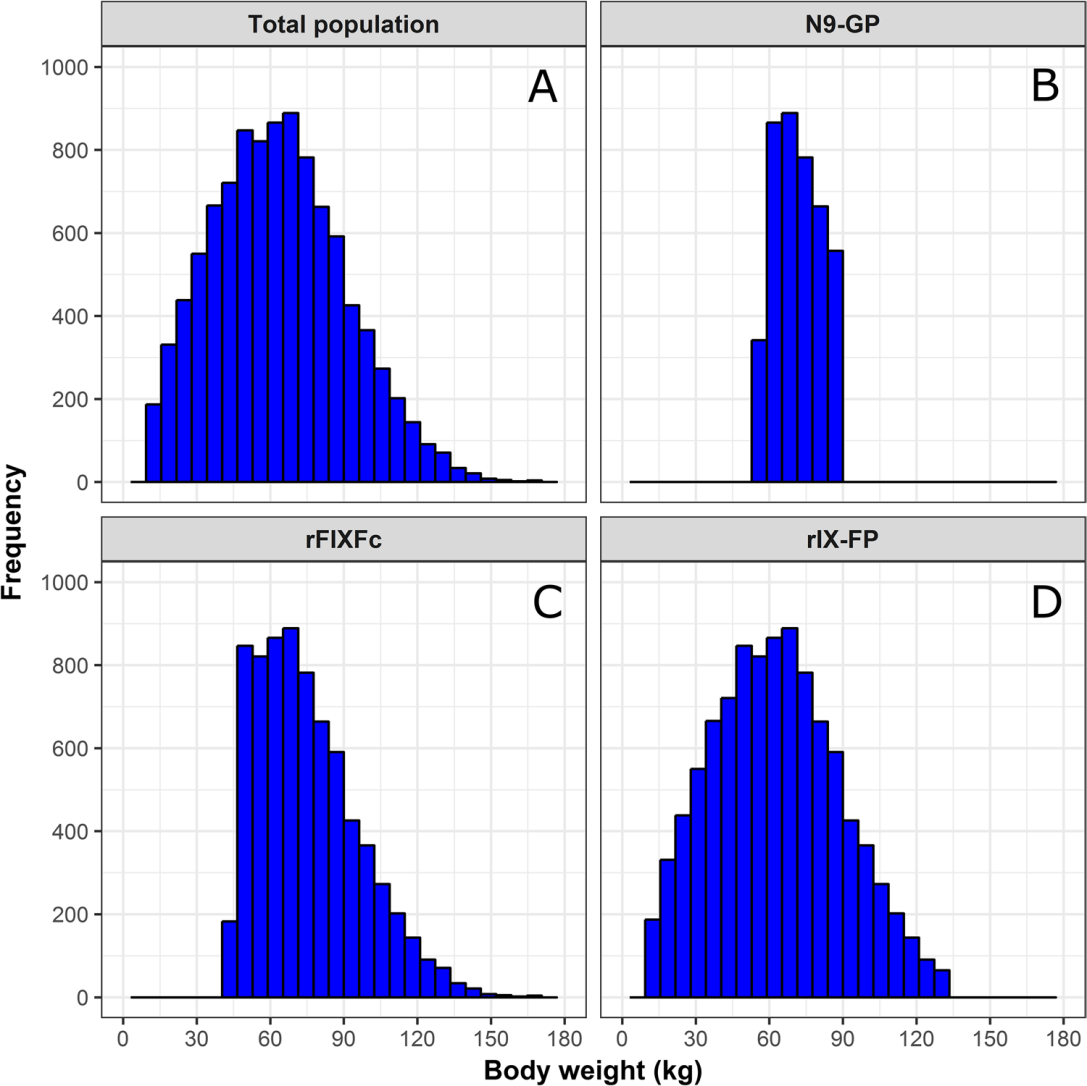
Distributions of the simulated body weights for the N9-GP, rFIXFc, and rIX-FP population. Histograms representing the body weight distributions in the total patient group (*n* = 10,000) and in the three patient selections made for the three population PK models (N9-GP: *n* = 4100, rFIXFc: *n* = 7290, rIX-FP: *n* = 9920). The body weight ranges were 56 to 90 kg, 45 to 187 kg, and 11 to 132 kg for N9-GP, rFIXFc, and rIX-FP, respectively

**Fig. 2:**
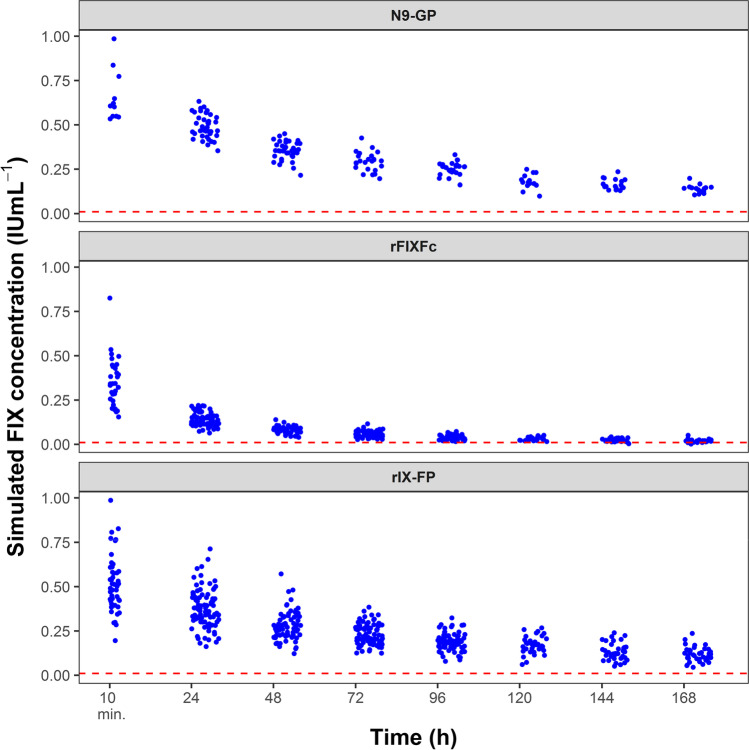
Simulated concentration–time curves of the three population PK models. The concentration–time data simulated using the three population PK models. The sequential observable data groups represent the consecutive sampling days from Table 2. The red dashed line depicts the lower limit of quantification (LLOQ: 0.01 IU mL^−1^)

A population of 10,000 virtual patients was simulated with a normal distribution of body weight (Fig. 1a). From this population, patients were selected with body weight ranges similar to the body weight ranges used to construct the respective population PK models (Fig. 1b–d). The number of patients selected for N9-GP, rFIXFc, and rIX-FP was 4100, 7290, and 9920, respectively. In Fig. 2, for each EHL-FIX product and each population, the FIX activity versus time profiles are shown, as obtained by Monte Carlo simulation using the corresponding population PK model from literature.

## N9-GP

In Table 3, the predictive performance of the LSSs for N9-GP is shown. In none of the virtual patients FIX levels were below the quantification limit (BLQ).

**Table 3:** Predictive performance for the N9-GP model

		Clearance			Terminal elimination half-life			Time until 1%			Calculated dose		
LSS	BLQ (%)	rMPE (%) [95% CI]		rRMSE (%)	rMPE (%) [95% CI]		rRMSE (%)	rMPE (%) [95% CI]		rRMSE (%)	rMPE (%) [95% CI]		rRMSE (%)
1	0	−0.1	[−0.4–0.1]	8.8	−1.2	[−1.7– − 0.8]	15.4	1.4	[1–1.7]	11.1	−1.3	[−1.7– − 0.9]	12.7
2	0	0.2	[0–0.4]	5.4	−0.1	[−0.6–0.4]	15.3	0.9	[0.6–1.1]	8.2	0	[−0.2–0.2]	6.1
3	0	0.7	[0.5–0.9]	5.5	−1.8	−2.4– − 1.3]	16.7	−0.2	[−0.5–0]	8.3	−0.4	[−0.5– − 0.2]	6
4	0	0.9	[0.8–1.1]	5.1	−2.1	[−2.6– − 1.6]	16.8	−0.6	[−0.9– − 0.4]	7.7	0	[−0.1–0.2]	4.9
5	0	1.4	[1.3–1.6]	5.2	−3.1	[−3.6– − 2.6]	16.7	−1.1	[−1.3– − 0.9]	7.3	0.1	[0–0.3]	4.4
6	0	0.5	[0.2–0.9]	11.8	−1.5	[−2– − 1]	16.1	1.4	[1–1.8]	13.2	−0.5	[−1–0]	17
7	0	−0.7	[−0.9– − 0.4]	8.4	−0.6	[−1.1– − 0.1]	15.7	2	[1.6–2.3]	11.1	−1.9	[−2.3– − 1.5]	12.6
8	0	−0.5	[−0.6– − 0.3]	6.4	−1.4	[−1.9– − 1]	15.5	1.2	[0.9–1.5]	9.3	−2.2	[−2.5– − 2]	9
9	0	0.4	[0.2–0.5]	5.4	−2.1	[−2.6– − 1.6]	15.3	0.4	[0.1–0.6]	8.2	−1.3	[−1.5– − 1.1]	6.4
10	0	0.6	[0.2–0.9]	11.9	−1.6	[−2.1– − 1.1]	16.3	1.3	[0.9–1.7]	13.2	−0.6	[−1.2– − 0.1]	17
11	0	0	[−0.2–0.2]	7.5	−0.6	[−1– − 0.1]	15.5	1.3	[1–1.6]	9.9	−0.7	[−1– − 0.4]	10.2
12	0	1.4	[1.2–1.6]	5.6	−3.2	[−3.7– − 2.7]	16.6	−1.3	[−1.5– − 1]	7.8	0.2	[0.1–0.4]	5.8
13	0	0	[−0.2–0.2]	6.4	0	[−0.4–0.5]	15.2	1.3	[1.1–1.6]	8.9	−0.2	[−0.4–0.1]	8.1
14	0	1.6	[1.4–1.7]	5.6	−3.4	[−3.9– − 2.9]	16.7	−1.2	[−1.5– − 1]	7.5	0.2	[0.1–0.4]	5.4

Predictive performance for 4100 virtual patients. *LSS* limited sampling schedule, *BLQ* below limit of quantification, *rMPE (*= *bias)* relative mean prediction error, *rRMSE (*= *precision)* relative root-mean-square error, *CI* confidence interval

The bias of the estimated PK parameters was relatively small for every evaluated LSS. All rMPEs were within the tolerance levels of −5% and 5%; the largest absolute bias was −3.4% for the *t*_1/2_, as obtained using LSS 14. However, the CL was consistently slightly overestimated for most of the LSSs, as can be seen from the rMPE 95% confidence intervals (95% CIs). Seemingly, this led to a slight underestimation of the *t*_1/2_ in most cases.

For each parameter, the precision from each LSS was acceptable with rRMSE values ranging from 4.4% to 17%. The precision was lowest for the *t*_1/2_, as compared to the other parameters. As the rMPE and the rRMSE were both within the tolerance levels, all LSSs can be applied. Nevertheless, LSS 5 showed the best overall predictive performance.

**Fig. 3:**
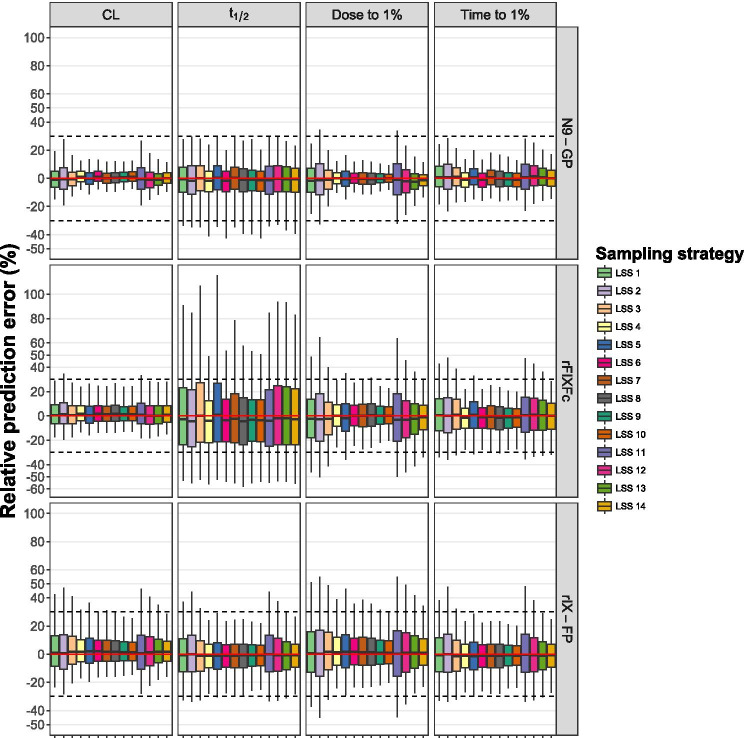
Relative prediction errors of the individual PK parameter estimates. Boxplots of the relative prediction errors (rPEs) from the different LSSs for the three population PK models. The extremities of the whiskers represent the 2.5% and 97.5% quantiles, the extremities of the boxes represent the 25% and 75% quantiles, and the black lines inside the boxes represent the modes of the rPE range. The red line represents zero and the black dashed lines represent −30% and 30% for the rPE range

In Fig. 3, it is shown that all rPEs for all PK parameters were within the −30% to 30% range, except for *t*_1/2_ using all LSSs and Dose_1%_ using LSS 2 or LSS 11.

### rFIXFc

In Table 4, the predictive performance of the LSSs for rFIXFc is shown. The percentage of observed samples BLQ depended on the applied LSS and ranged from 0 to 13%.

**Table 4:** Predictive performance for the rFIXFc model

		Clearance			Terminal elimination half-life			Time until 1%			Calculated dose		
LSS	BLQ (%)	rMPE (%) [95% CI]		rRMSE (%)	rMPE (%) [95% CI]		rRMSE (%)	rMPE (%) [95% CI]		rRMSE (%)	rMPE (%) [95% CI]		rRMSE (%)
1	0	2.1	[1.8–2.4]	12	2.9	[2–3.7]	37.7	1.6	[1.1–2]	19.8	−1.6	[− 2.1– − 1]	24
2	5	2.3	[2–2.5]	10.1	0.7	[−0.1–1.6]	36	−1.2	[−1.5– − 0.8]	14.3	0.5	[0.1–0.8]	15.2
3	5	2.9	[2.6–3.1]	10.7	−3.5	[−4.2– − 2.7]	30.6	−2.2	[−2.5– − 1.8]	14	0.9	[0.6–1.3]	15.3
4	11.4	2.5	[2.3–2.7]	9.7	−3.2	[−3.9– − 2.5]	27.8	−1.9	[−2.2– − 1.6]	12.4	0.6	[0.3–0.9]	13
5	12.5	2.6	[2.4–2.9]	9.8	−3.3	[−4– − 2.7]	26.9	−2	[−2.3– − 1.7]	11.9	0.6	[0.3–0.8]	12
6	0	2.7	[2.4–3]	13.5	0.9	[0–1.7]	36.1	1.8	[1.3–2.3]	21.5	−0.2	[−0.9–0.5]	29.3
7	0	1.5	[1.2–1.7]	11.9	3.5	[2.7–4.4]	38.4	1.9	[1.5–2.4]	19.8	−2.4	[−2.9– − 1.8]	23.5
8	0.2	1.7	[1.4–1.9]	11.7	3.4	[2.5–4.3]	39	0.9	[0.5–1.3]	17.9	−2.6	[−3– − 2.1]	19.6
9	1.4	2.4	[2.2–2.7]	11.2	1.4	[0.6–2.2]	35.7	−0.5	[−0.9– − 0.2]	15.5	−1.3	[−1.7– − 0.9]	16
10	0	3	[2.6–3.3]	14	0.5	[−0.3–1.3]	36.1	1.6	[1.1–2.1]	21.5	0	[−0.6–0.7]	29.4
11	0.2	1.6	[1.3–1.8]	11.7	6.7	[5.7–7.7]	42.2	1.6	[1.1–2]	18.3	−1.9	[−2.3– − 1.4]	20.6
12	11.3	2.8	[2.6–3.1]	10	−4.4	[−5– − 3.7]	27.1	−2	[−2.3– − 1.7]	12.8	0.7	[0.4–1]	15.3
13	1.3	1.8	[1.5–2]	11	7.5	[6.5–8.5]	44.7	0.8	[0.4–1.2]	16.5	−1.2	[−1.6– − 0.8]	18
14	11.3	2.7	[2.5–3]	10.3	−3	[−3.6– − 2.3]	27.5	−1.8	[−2.1– − 1.4]	12.7	0.6	[0.3–0.9]	14.3

Predictive performance for 7290 virtual patients. *LSS* limited sampling schedule, *BLQ* below limit of quantification, *rMPE (*= *bias)* relative mean prediction error, *rRMSE (*= *precision)* relative root-mean-square error, *CI* confidence interval

The rMPE values of CL, Time_1%_, and Dose_1%_ were within the tolerance interval of −5% to 5%. The rMPE values for *t*_1/2_ were generally adequate with an exception for LSS 11 and 13 (LSSs with the last sample on day 5) with respective values of 6.7 and 7.5%. Every LSS resulted in a slight overestimation of the CL, as shown by the 95% CIs. However, there is no consistent pattern of overestimation or underestimation for the other parameters.

While the rRMSE values for the CL and Time_1%_ were below 25%, the precision of the Dose_1%_ was too low for LSS 6 and LSS 10 (LSSs with the last sample on day 3) with respective values of 29.3% and 29.4%. The precision of the *t*_1/2_ estimates turned out to be problematic for all of the LSSs; with the rRMSEs ranging from 27.1% to 44.7%. It is noteworthy that the predictive performance of LSSs containing samples after the seventh sampling day (LSS 4, 5, 12, 14) was superior compared to the predictive performance of other LSSs. Overall, LSS 5 showed the best predictive performance with samples taken on days 1, 5, 7, and 8.

Although the rPEs of CL using LSS 2 and 11 were slightly outside the −30% and 30% range, the other LSSs showed an acceptable rPE range (Fig. 3). The error ranges for t_1/2_ were all outside the acceptable range, which was also shown by the low precision of t_1/2_ for each LSS. LSS 4, 6, 7, 8, 9, 10, and 14 produced rPEs within the −30% to 30% range for CL, Time1%, and Dose_1%_.

### rIX-FP

In Table 5, the predictive performance of the LSSs for rIX-FP is shown. For each of the LSSs, less than 1% of the patients had a FIX level BLQ.

**Table 5:** Predictive performance for the rIX-FP model

		Clearance			Terminal elimination half-life			Time until 1%			Calculated dose		
LSS	BLQ (%)	rMPE (%) [95% CI]		rRMSE (%)	rMPE (%) [95% CI]		rRMSE (%)	rMPE (%) [95% CI]		rRMSE (%)	rMPE (%) [95% CI]		rRMSE (%)
1	0	3.4	[3.1–3.8]	17.4	0	[−0.4–0.3]	17.9	0.1	[− 0.2–0.5]	18.2	2.6	[2.1–3]	23
2	0.01	3.2	[3–3.5]	12.6	−1.4	[−1.6– − 1.1]	13.3	−1.4	[−1.7– − 1.2]	12.9	2.9	[2.5–3.2]	16
3	0.01	3	[2.8–3.3]	12.1	−1.3	[−1.5– − 1]	13.4	−1.3	[−1.6– − 1.1]	12.8	2.5	[2.2–2.8]	15.2
4	0.02	2.5	[2.3–2.7]	10.9	−1	[−1.3– − 0.8]	12.2	−1.1	[−1.3– − 0.9]	11.6	2	[1.7–2.2]	13.6
5	0.02	2.5	[2.3–2.7]	10.5	−1.1	[−1.3– − 0.8]	11.7	−1.1	[−1.4– − 0.9]	11	2	[1.7–2.2]	12.8
6	0	2.8	[2.5–3.2]	19.2	1.3	[0.9–1.7]	19.9	1.7	[1.2–2.1]	20.9	1.3	[0.8–1.8]	25.1
7	0	3.3	[3–3.6]	16.6	0	[−0.3–0.4]	17.9	0.1	[−0.2–0.5]	18	2.3	[1.8–2.7]	21.9
8	0	3.3	[3.1–3.6]	13.9	−0.9	[−1.2– − 0.6]	15.5	−1	[−1.3– − 0.7]	15.1	2.6	[2.3–3]	18
9	0	2.9	[2.6–3.1]	11.8	−1	[−1.3– − 0.8]	13.6	−1.1	[−1.4– − 0.9]	12.9	2.2	[1.9–2.5]	14.8
10	0	2.9	[2.5–3.3]	19.5	1.4	[1–1.7]	20	1.7	[1.3–2.1]	20.9	1.3	[0.8–1.8]	25.3
11	0	4	[3.7–4.3]	16.2	−1	[−1.3– − 0.6]	16.5	−1	[−1.3– − 0.6]	16.5	3.5	[3–3.9]	21.4
12	0.02	3.2	[2.9–3.4]	12.7	−1.3	[−1.6– − 1.1]	13.1	−1.4	[−1.6– − 1.1]	12.8	2.8	[2.5–3.1]	16.2
13	0	3.7	[3.4–3.9]	14.8	−1.2	[−1.5– − 0.9]	15	−1.2	[−1.5– − 0.9]	14.9	3.2	[2.8–3.6]	19.3
14	0.02	3.1	[2.9–3.4]	12.3	−1.3	[−1.6– − 1.1]	12.8	−1.4	[−1.7– − 1.2]	12.3	2.8	[2.5–3.1]	15.4

Predictive performance for 9920 virtual patients. *LSS* limited sampling schedule, *BLQ* below limit of quantification, *rMPE (*= *bias)* relative mean prediction error, *rRMSE (*= *precision)* relative root-mean-square error, *CI* confidence interval

Except for LLS 6 and LSS 10, all of the LSSs had an adequate bias and precision. The rMPEs of the four PK parameters were within the −4% and 4% range for every LSS. None of the 95% CIs for the rMPE of CL and Dose_1%_ contained zero, indicating that every LSS resulted in a slight overestimation of these parameters. Apparently, according to the 95% CIs, this did lead to a slight underestimation of *t*_1/2_ and Time_1%_ in some of the LSSs.

For all LSSs, the precision was acceptable for all PK parameters, except for the calculated Dose_1%_ using LSS 6 and 10. However, the precision was substantially higher for LSSs containing a sample taken after the fifth day (LSS 2, 3, 4, 5, 9, 12, 13, 14). Overall, LSS 4 showed the best predictive performance. As LSS 6 and LSS 10 led to precision values > 25% for the calculated Dose_1%_, these LSSs are not recommended.

Although the rPE ranges for CL, time_1%_, and t_1/2_ were mostly within the acceptable ranges, large ranges were obtained for the Dose_1%_. LSS 2 and LSS 11 showed the least predictive performance in terms of rPE, as ranges were outside the acceptable limits for each of the PK parameters CL, *t*_1/2_, and Dose_1%_.

## Discussion

In this study, the predictive performance of 14 LSSs was assessed with regard to their ability to adequately estimate individual PK parameters from the population PK models of three currently available EHL-FIX concentrates. To determine the number of samples and the time of blood sampling for clinical practice, Bayesian analysis of simulated concentration–time curves was performed. For N9-GP, rIX-FP, and rFIXFc, bias and precision for CL and Time_1%_ from all LSSs were acceptable. Acceptable bias and precision of *t*_1/2_ were found for all LSSs of N9-GP and rIX-FP. For rFIXFc, the precision of t_1/2_ was unsatisfactory. Moreover, for all EHL-FIX products, bias and precision of Dose_1%_ were acceptable for all LSSs, except for LSS with the last sample taken on day 3 (LSS 6, 10). Best predictive performance based on bias and precision was demonstrated for N9-GP, rFIXFc, and rIX-FP by LSS 5, LSS 5, and LSS 4 with samples taken on days 1, 5, 7, and 8 and on days 1, 4, 6, and 8, respectively.

This study showed that for N9-GP all LSSs demonstrated an adequate predictive performance, with the rMPE smaller than 5%, the rRMSE smaller than 25%, and the rPE range between −30% and 30% for all estimated PK parameters. As suggested above, with the current treatment targets each of the investigated LSSs could be clinically applicable. For rFIXFc, none of the LSSs had an acceptable rRMSE or rPE range for the *t*_1/2_. However, except for LSS 6 and LSS 10, all LSSs showed an acceptable predictive performance for the other parameters. Therefore, these LSSs could still be applied in the clinical setting, as Time_1%_ and Dose_1%_ could still be estimated accurately. For all EHL-FIX products, lower predictive performance was obtained with sampling only until day 3 (< 56 h), as LSS 6 and LSS 10 did not show acceptable bias and precision for Dose_1%_. In clinical practice, LSSs should be applied which contain samples taken after day 3 and, preferably, on day 8.

In the 8-day sample period, virtual patients receiving rFIXFc exhibited the highest percentage of FIX levels BLQ with a value of 13% for LSS 5. Patients receiving N9-GP and rIX-FP practically did not have FIX levels BLQ. If a sample with activity BLQ is obtained, it is advised to use an LSS with sampling times closer to dose administration. As LSS 5 was the most preferable LSS for rFIXFc, LSS 3 might be applied instead showing only slightly less predictive performance.

Elimination half-life is determined by clearance (e.g., CL, Q, Q2) and volumes of distribution (e.g., V1, V2, V3). Thereby, inter-patient variability in these parameters will produce inter-patient variability in *t*_1/2_. As large rPEs were obtained for the *t*_1/2_ from the population PK model for rFIXFc, these are most likely due to having inter-patient variability specified for the volume of distribution for the second (V2) and third (V3) compartments and the inter-compartmental clearance between the first and the second compartment (Q2), besides from clearance (CL) and volume of distribution (V1) from the central compartment. Allowing large inter-patient variability for the population PK parameters reduces the amount of information supplied a priori in Bayesian analysis. Therefore, these large variabilities may lead to diminished predictive performance for the estimation of individual PK parameters. This is especially true in a sparse sampling situation, which is often encountered in clinical practice. In contrast, for the population PK model of N9-GP, a large IIV was specified for Q (127.3%). However, this large IIV did not lead to unacceptable rPE ranges for each of the parameters. As a result, similar sampling times for different products may lead to different results and LSSs should, therefore, be specified for each product separately.

In the published population PK model for rFIXFc, endogenous baseline FIX levels were subtracted from the observed FIX levels [15]. Furthermore, observed levels were corrected for potential incomplete washout. As a result, the population model describes the PK in severe hemophilia B patients. The published N9-GP model assumed that the endogenous baseline FIX level was zero, whereas for the rIX-FP model the endogenous baseline level was estimated and consequently subtracted from the observed FIX levels (Table 1) [14, 16]. Concluding, all models can be used to simulate FIX levels for severe hemophilia B patients, as performed in the present study. Not simulating any endogenous baseline FIX levels in the present study has not influenced the results obtained for the three compounds. Moreover, these LSS may also be applicable in moderate hemophilia B patients.

In several studies [9, 24, 25], it has been suggested that the increased size of the EHL-FIX molecule with respect to the SHL-FIX molecule causes a change in its distribution into the extravascular compartment. Furthermore, it was proposed that extravascular FIX, bound to collagen IV on endothelial cells, is important for long-lasting hemostatic protection [9, 25]. In this case, with the current target trough levels of 1% during prophylactic treatment using EHL-FIX concentrates, sufficient hemostatic protection may not be guaranteed. However, the EHL-FIX concentrates proved to be efficacious in the prevention and treatment of bleeds in clinical trials [26, 27]. However, it should be realized that trough levels much higher than 1% were achieved in these studies. Therefore, the effect of the change in extravascular distribution remains unknown.

In a real-life setting, the patient population in which Bayesian analysis is applied must be similar to the population in which the population model has been constructed. In the Bayesian estimation procedure, the population PK parameters are used derived from the population used for the construction of the model as a priori information. Therefore, if the patient population to which this model is applied, differs from the population used for the construction of these models (Table 1), then the a priori information of the Bayesian estimation might be biased. This could lead to a significant bias for the estimated individual PK parameters. For instance, when Bayesian analysis is applied in children using a population PK model constructed with data from adults, the individual parameter estimates (e.g., CL) may be underestimated, as it has been reported that (weight-normalized) clearance in children is higher than in adults [28].

This study was performed *in silico* and, therefore, real-world validation is still required before incorporation of the established LSSs into guidelines. The results presented here can be, however, used for the design of such a validation study. In this study, it was assumed that the parameters for which the predictive performance was evaluated, yield sufficient information to perform patient-tailored dosing, as the current goal of prophylaxis is to maintain FIX plasma levels above 1%. It is, however, not yet known if this target guarantees the efficacy of prophylactic dosing using FIX concentrates [29]. If it does not, then the estimation of different parameters may be necessary. However, prophylaxis with a target trough level above 1% has proven to be an effective treatment strategy in long-term follow-up studies [29].

## Conclusion

In this *in silico* study, several LSSs were proposed and their ability to estimate individual PK parameters for three currently licensed EHL-FIX concentrates was evaluated. Any of the LSSs proved to have adequate predictive performance for the population PK model for N9-GP. For the population PK model for rFIXFc, every LSS turned out to be inappropriate to estimate *t*_1/2_ with adequate precision. Moreover, LSS without a sample taken after day 3 (LSS 6 and LSS 10) cannot be recommended for rFIXFc and rIX-FP, as the Dose_1%_ could not be obtained accurately. Best predictive performance was demonstrated for N9-GP, rFIXFc, and rIX-FP by LSS 5, LSS 5, and LSS 4, with samples taken on days 1, 5, 7, and 8 and on days 1, 4, 6, and 8, respectively. Whether the obtained LSSs are adequate in a real-world setting remains to be validated through further studies. The results from this study may be used to design such clinical trials.

## Data Availability

All data and material can be obtained upon any reasonable request at r.mathot@amsterdamumc.nl.
